# The Prognostic Effect of Circadian Blood Pressure Pattern on Long-Term Cardiovascular Outcome Is Independent of Left Ventricular Remodeling

**DOI:** 10.3390/jcm8122126

**Published:** 2019-12-02

**Authors:** Marijana Tadic, Cesare Cuspidi, Vera Celic, Biljana Pencic, Giuseppe Mancia, Guido Grassi, Goran Stankovic, Branislava Ivanovic

**Affiliations:** 1 Department of Internal Medicine and Cardiology, Charité—Universitätsmedizin Berlin, Campus Virchow-Klinikum, Augustenburgerplatz 1, 13353 Berlin, Germany; 2 Clinical Research Unit, University of Milan-Bicocca and Istituto Auxologico Italiano IRCCS, Viale della Resistenza 23, 20036 Meda, Italy; 3 Department of Cardiology, University Clinical Hospital Center “Dr. Dragisa Misovic—Dedinje”, Heroja Milana Tepica 1, 11000 Belgrade, Serbia; 4 Department of Medicine and Surgery, Clinica Medica, University Milano-Bicocca, 20126 Milan, Italy; 5 Clinic of Cardiology, Clinical Center of Serbia, Koste Todorovic 8, 11000 Belgrade, Serbia

**Keywords:** hypertension, left ventricle, hypertrophy, diastolic dysfunction, non-dipping, reverse dipping

## Abstract

We aimed to investigate the predictive value of 24 h blood pressure (BP) patterns on adverse cardiovascular (CV) outcome in the initially untreated hypertensive patients during long-term follow-up. This study included 533 initially untreated hypertensive patients who were involved in this study in the period between 2007 and 2012. All participants underwent laboratory analysis, 24 h BP monitoring, and echocardiographic examination at baseline. The patients were followed for a median period of nine years. The adverse outcome was defined as the hospitalization due to CV events (atrial fibrillation, myocardial infarction, myocardial revascularization, heart failure, stroke, or CV death). During the nine-year follow-up period, adverse CV events occurred in 85 hypertensive patients. Nighttime SBP, non-dipping BP pattern, LV hypertrophy (LVH), left atrial enlargement (LAE), and LV diastolic dysfunction (LV DD) were risk factors for occurrence of CV events. However, nighttime SBP, non-dipping BP pattern, LVH, and LV DD were the only independent predictors of CV events. When all four BP pattern were included in the model, non-dipping and reverse dipping BP patterns were associated with CV events, but only reverse-dipping BP pattern was independent predictor of CV events. The current study showed that reverse-dipping BP pattern was predictor of adverse CV events independently of nighttime SBP and LV remodeling during long-term follow-up. The assessment of BP patterns has very important role in the long-time prediction in hypertensive population.

## 1. Introduction

The influence of arterial hypertension on left ventricular (LV) structural and functional remodeling is well known [1,2]. There are numerous hemodynamic and biohumoral mechanisms that connect hypertension with LV hypertrophy (LVH) and LV diastolic dysfunction (LV DD) [1,2]. LVH and LV DD are significantly associated with cardiovascular (CV) morbidity and mortality in the hypertensive population [3,4,5,6].

Circadian blood pressure (BP) patterns have important impact on LV structural and functional changes (LVH and LV DD [7]), as well as CV outcome in hypertensive population [8]. Non-dipping BP pattern was shown as important predictor of CV morbidity and mortality [9]. Updated classification of circadian BP patterns that involves extreme and reverse dippers raised the question regarding the importance of extreme and reverse dipping.

Our study group recently reported that extreme dipping was not invariably associated with nighttime hypotension as it was considered before [10]. Furthermore, our pooled analysis revealed no difference in LV mass index between extreme dippers and dippers [11]. However, LV mass index was significantly higher in non-dippers and reverse dippers compared to extreme dippers [11]. Similar analysis demonstrated that the risk of nonfatal and fatal cardiovascular events in patients with reverse dipping BP pattern was 2.5-fold greater than in dippers and 2.1-fold higher than in non-dippers [12].

The aim of the present study was to investigate predictive effect of circadian BP patterns and LV remodeling on occurrence of CV events in initially untreated hypertensive patients during a nine-year follow-up period.

## 2. Materials and Methods

This follow-up study included 587 recently diagnosed untreated hypertensive subjects referred to outpatient clinic of Clinical Centre of Serbia and University Hospital “Dr. Dragisa Misovic—Dedinje” due to 24 h ambulatory BP monitoring (ABPM) in the period between 2007 and 2013. The inclusion criteria were age (≥18 years) and untreated arterial hypertension confirmed by ABPM. Subjects with symptoms and signs of heart failure, coronary artery disease, previous cerebrovascular insult, atrial fibrillation, congenital heart disease, significant valve heart disease, neoplastic disease, renal failure, sleep apnea, morbid obesity, or endocrinology diseases, including diabetes mellitus, were excluded from the study. Antihypertensive therapy was started immediately after diagnosis of arterial hypertension was confirmed. However, there are no data on how antihypertensive therapy was changed and adjusted during follow-up period. Anthropometric measures and laboratory analyses were taken from all study participants. Body mass index (BMI) and body surface area (BSA) were calculated for each patient. In the follow-up, 54 patients were lost. The study was approved by the local Ethics Committee (MR/3289/2007), and informed consent was obtained from all the participants.

### 2.1. BP Measurement

Clinic BP was measured in the morning hours and calculated as the average value of the two consecutive measurements in the sitting position taken 5 min apart. BP was obtained in at least two different occasions. All patients underwent a 24 h ambulatory BP monitoring (ABPM) using a Schiller BR-102 plus system (Schiller AG, Baar, Switzerland). The device was programmed to measure BP at 20-min intervals during the day (07:00–23:00) and at 30-min intervals during the night (23:00–07:00). The recording was then analyzed to obtain a 24 h daytime and nighttime average systolic BP (SBP), diastolic BP (DBP), and heart rate. The patients performed their usual daily activities. Nighttime BP was defined as the average of BPs from the time when the patients went to bed until the time they got out of the bed, and daytime BP as the average of BP recorded during the rest of the day. Arterial hypertension was defined as 24 h SBP ≥130 mmHg and/or DBP ≥80 mmHg [13].

The nocturnal dipping was defined as a reduction in average SBP at night ≥10% and <20% compared with average daytime values; the extreme nocturnal dipping existed if the reduction in average SBP at night was ≥20% [14,15]. The non-dippers had a nocturnal reduction in average SBP <10%, and the reverse dippers were the patients with higher nocturnal average SBP in comparison with diurnal values [14,15].

### 2.2. Echocardiography

Echocardiographic examination was performed by Acuson Sequoia 256 ultrasound system (Siemens AG, Erlangen, Germany) and Vivid 7 ultrasound machine (GE Healthcare, Horten, Norway). The values of all echocardiographic parameters were calculated as the average value of three consecutive cardiac cycles. LV diameters, interventricular septum, and relative wall thickness were determined according to the recommendations [16]. LV ejection fraction (EF) was assessed by the biplane method. LV mass was calculated by using the formula of the American Society of Echocardiography [16] and indexed for body surface area. LV hypertrophy (LVH) was defined as LV mass index ≥95 g/m^2^ in women and ≥115 g/m^2^ in men [16]. Left atrial (LA) volume was measured by the biplane method in four- and two-chamber views and indexed for BSA (LAVI). LA enlargement (LAE) was defined as LAVI >34 mL/m^2^ [16]. However, volumetric LA measurements were available in the half of population.

Pulsed-wave Doppler in the apical four-chamber view was used for the evaluation of early and late transmitral diastolic flow velocities (E and A, respectively) and deceleration time (DT) [17]. Tissue Doppler imaging was used to get LV myocardial velocities in the apical four-chamber view, with a sample volume placed at the septal and lateral segments of the mitral annulus during early diastole (e’). The average of the peak early diastolic relaxation velocity (e´) of the septal and lateral mitral annulus was obtained, and the E/e´ ratio was computed [17]. LV diastolic dysfunction (LV DD) was defined by the presence of at least 50% of following criteria: E/e’ >14, septal e’ <7 cm/s or lateral e’ <10 cm/s, tricuspid regurgitation velocity >2.8 m/s, and LAVI >34 mL/m^2^ [18]. At least three criteria should be available in order to assess LV DD.

### 2.3. Follow-Up

The primary outcome included fatal and non-fatal CV events (atrial fibrillation, myocardial infarction, myocardial revascularization, heart failure, stroke, and CV death). All CV events during follow-up period were defined according to the International Classification of Diseases (ICD) code. Follow-up was performed during 2019. The information regarding CV events was obtained directly from the patients or from their local general practitioners or cardiologists. All events that we investigated in the follow-up required hospitalization and therefore the majority of patients who had these CV events had a discharge letter from the regional hospital with adequate ICD code for each diagnosis. However, the information obtained from the local general practitioners and cardiologists could not show when the event occurred, which had some consequence in the choice of statistical tests.

### 2.4. Statistical Analysis

The data were analyzed by using SPSS version 21 (SPSS, Inc, Chicago, IL, USA). Continuous variables were presented as the mean ± standard deviation (SD) and were compared by using the t-test for two independent samples as they showed a normal distribution. The differences in proportion were compared by using the χ² test. Univariate and multivariate logistic regression analyses were used for determination of association between different parameters of LV structure, LV function, and 24 h BP patters and CV events. The primary outcome included atrial fibrillation, myocardial infarction, myocardial revascularization, heart failure, stroke or CV death. Model 1 used BP pattern as dichotomous variable (dipping/non/dipping), whereas Model 2 involved all four 24 h BP patterns (dipping/non-dipping/extreme dipping/reverse dipping). Both models did not include LAE in multivariate analysis because LAE represents one of the criteria for LV DD that was used in this model. The *p*-value < 0.05 was considered statistically significant.

## 3. Results

The primary outcome was detected in 85 hypertensive patients (24 atrial fibrillation, 12 myocardial infarction, 16 myocardial revascularization without infarction, 14 heart failure, 10 strokes, and 9 CV death). Patients who experienced some CV events were older and more obese than patients without CV event (Table 1). There was no difference in gender distribution between two groups. Plasma glucose was similar between the observed groups, but patients with CV event had higher level of total cholesterol, triglycerides and creatinine. Supplementary Table S1 showed the main clinical parameters and 24 h ABPM for all four BP patterns in the study population.

Regarding 24 h BP patterns, reverse dippers were more prevalent among patients who had some CV event than in the group without CV events (Table 1). There was no significant difference in the prevalence of other BP patterns between two groups.

Clinic, 24-h BP, daytime and nighttime BP values (systolic and diastolic), as well as corresponding heart rate, were higher in the hypertensive patients who had CV event during the follow-up period (Table 1).

### 3.1. Echocardiographic Measurements

LV diameter was similar between two groups (Table 2). Parameters of LV hypertrophy (wall thickness, mass index) were significantly higher in patients with CV event (Table 2). The prevalence of LVH was also significantly higher in this group of patients. LAVI was higher in patients with CV event and the prevalence of LAE was significantly higher in this group (Table 2). E/A ratio was significantly lower, E/e’ ratio significantly higher, and DT significantly longer in hypertensive patients who had CV event (Table 2). LV DD was significantly more prevalent in the patients with CV event.

### 3.2. Logistic Regression Analyses

Univariate logistic regression analysis in Model 1 revealed that age, total cholesterol level, nighttime SBP, LVH, LAE, LV DD, and non-dipping BP pattern (including non-dipping and reverse BP patterns) were associated with occurrence of CV event during the follow-up. Multivariate analysis showed that only nighttime SBP, LVH, LV DD, and non-dipping BP pattern were independent predictors of CV events in the whole study population.

Univariate logistic regression analysis in Model 2 revealed that both, non-dippers and reverse dippers were related with higher occurrence of CV events, besides other aforementioned parameters (Table 3). However, only reverse dipping BP pattern was independently associated with CV event occurrence (Table 3).

## 4. Discussion

The present study provided several important findings that deserve further discussion—(i) non-dipping and reverse dipping BP patterns were related with CV events; (ii) only reverse dipping BP was independently of LV structural and functional remodeling related with adverse outcome; and (iii) nighttime SBP was independently associated with CV events.

Our study group showed that 24 h BP patterns had important effect on LV and right ventricular remodeling in untreated hypertensive patients [7,19,20]. Other investigators emphasized the negative impact of non-dipping BP pattern on CV morbidity and mortality [8]. Nevertheless, data regarding predictive value of new BP patterns—reverse and extreme dipping are still scarce. The present findings showed that non-dipping and reverse dipping BP patterns correlated with adverse CV outcome, unlike dipping and extreme dipping patterns. However, only reverse dipping pattern was independently of LVH, LV DD, and other CV risk factors associated with adverse CV events during the nine-year follow-up. Bastos et al. found reverse dipping BP pattern in 7% of participants and showed that reverse dippers had significantly higher risk of CV event and stroke than dippers during eight-year follow-up [21]. However, there was no difference compared to non-dippers. The important limitation is the fact that echocardiographic parameters such as LVH and LV DD were not available and therefore could not be included into the statistical models [21]. Kim et al. found reverse dipping in 27% of hypertensive patients and reported that reverse dippers had higher CV mortality than dippers and non-dippers during 10-year-follow-up [22]. Echocardiographic data were also not available and effect of LVH and LV DD in the same model with BP patterns could not be determined [22]. Our pooled analysis showed that the risk of nonfatal and fatal CV events in patients with reverse dipping pattern was 2.5-fold greater than in dippers (95% CI, 2.11–2.96, *p* < 0.01) and persisted to be 2.1-fold higher compared with non-dipping pattern (95% CI, 1.77–2.45, *p* < 0.001) [12].

Our study revealed that nighttime SBP was independently of LVH, LV DD and BP patterns associated with adverse CV outcome during long-term follow-up. This could be related with increased risk of cardiac and vascular changes in patients with nighttime hypertension that was reported in our meta-analysis [23]. Furthermore, the large study that involved 7458 participants with 24 h ABPM demonstrated that nighttime ambulatory BP was more closely associated with fatal and nonfatal cardiovascular events (stroke, myocardial infarction, and cardiovascular death) than daytime ambulatory BP during a median follow-up of 9.6 years [24].

The possible mechanisms that might clarify negative influence of reverse dipping BP pattern on CV outcome is sympathetic nervous system (SNS). Significantly higher SNS activation has already been reported in reverse dippers compared to other BP patterns [25]. The lowest sympathetic activity was found in extreme dippers but without statistically significant difference in comparison with dippers and non-dippers [25].

The main advantage of our study is involvement of young, initially untreated low-risk hypertensive patients, as well as detailed evaluation of 24 h ABPM, LV structure, and systolic and diastolic function. We had the possibility to include all these BP and echocardiographic parameters in the statistical models, which ultimately showed that reverse dipping pattern was the only BP pattern that was associated with adverse CV events in hypertensive population independently of LV structure and function, age, gender, BMI, nighttime BP, creatinine, and cholesterol levels. On one side, studies were usually focused on predictive value of office BP and/or 24 h ABPM when assessed CV risk in hypertensive or general population, but not on BP patterns [26], whereas other investigations were more concentrated on BP patterns, without including detailed assessment of LV structure and function [23,24]. Additionally, our patients were initially free of comorbidities such as diabetes and renal insufficiency that could significantly impact the final results [27].

The main clinical implication of this study is that 24 h ABPM should be performed at the moment of diagnosis of arterial hypertension, even in the low-risk hypertensive patients because ABPM reveals BP patterns and patients with nighttime hypertension who are potentially at the higher risk for unfavorable CV outcome during long-term follow-up.

### Limitations

This study showed some limitations. (i) We included only uncomplicated and untreated hypertensive patients, which decreases potential generalization of our findings on patients with comorbidities. (ii) Systolic BP was used for classification of circadian BP patterns which also could have an influence on the final results. (iii) The lack of normotensive group is potential limitation because there was no possibility to compare outcome between hypertensive patients and initially normotensive subjects. (iv) The effect of antihypertensive therapy was not possible to determine because there are no data how therapy was changed during follow-up period. (v) Polysomnography was not performed and possibly the prevalence of sleep apnea could be higher in the study population. However, we excluded patients with clinical symptoms of sleep apnea, and we do not believe that real prevalence of sleep apnea in this young and low-risk hypertensive population could be high enough to impact final results and conclusions.

## 5. Conclusions

The present investigation showed that non-dipping and reverse dipping BP patters correlated with CV events during long-term follow-up. However, only reverse dipping was independently of nighttime SBP, LV remodeling, and other usual CV risk factors related with adverse CV outcome in initially untreated hypertensive patients. Our study emphasized the importance of timely performance of detailed echocardiographic examination and 24 h ABMP because parameters obtained with both methods and combination of these parameters could determine patients who are at higher risk for future CV events. Therefore, adverse CV events might be prevented or postponed with adequate therapeutic approach in these patients.

## Figures and Tables

**Table 1:** Demographic characteristics and clinical parameters of study population.

	Without CV Events (*n* = 448)	Adverse CV Events (*n* = 85)	*p*
Age (years)	51 ± 10	54 ± 12	0.015
Male (%)	237 (53)	48 (56)	0.627
BMI (kg/m^2^)	26.9 ± 4.1	28.1 ± 4.6	0.016
Plasma glucose (mmol/L)	5.2 ± 1.8	5.6 ± 1.7	0.059
Total cholesterol (mmol/L)	5.6 ± 2.1	6.5 ± 2.4	<0.001
Triglycerides (mmol/L)	1.8 ± 1.0	2.4 ± 1.2	<0.001
Serum creatinine (mmol/L)	92 ± 20	100 ± 25	0.001
24-h BP patterns			
Dippers (%)	189 (42)	25 (29)	0.037
Non-dippers (%)	134 (30)	34 (40)	0.088
Extreme dippers (%)	70 (16)	7 (8)	0.108
Reverse dippers (%)	55 (12)	19 (22)	0.022
Clinic			
SBP (mmHg)	151 ± 14	158 ± 16	<0.001
DBP (mmHg)	94 ± 9	97 ± 10	0.006
Heart rate (beat/min)	76 ± 12	79 ± 11	0.033
24 h			
SBP (mmHg)	137 ± 13	142 ± 14	0.001
DBP (mmHg)	81 ± 10	86 ± 11	<0.001
Heart rate (beat/min)	70 ± 9	73 ± 10	0.006
Daytime			
SBP (mmHg)	140 ± 14	144 ± 13	0.015
DBP (mmHg)	83 ± 11	88 ± 12	<0.001
Heart Rate (beat/min)	72 ± 10	75 ± 11	0.013
Nighttime			
SBP (mmHg)	125 ± 10	136 ± 12	<0.001
DBP (mmHg)	78 ± 8	82 ± 8	<0.001
Heart Rate (beat/min)	61 ± 7	66 ± 8	<0.001

BMI: body mass index; BP: blood pressure; SBP: systolic blood pressure: DBP: diastolic blood pressure.

**Table 2:** Echocardiographic parameters of left ventricular structure and function in the study population.

	Without CV Events (*n* = 448)	Adverse CV Events (*n* = 85)	*p*
LVEDD (mm)	48.8 ± 4.5	49.8 ± 4.7	0.063
IVS (mm)	9.7 ± 1.1	10.4 ± 1.3	<0.001
RWT	0.39 ± 0.08	0.41 ± 0.09	0.039
LVMI (g/m^2^)	86.3 ± 8.9	99.5 ± 12.7	<0.001
LVH (%)	67 (15)	27 (32)	<0.001
EF (%)	64 ± 4	63 ± 5	0.043
LAVI * (mL/m^2^)	29.5 ± 4.7	33.4 ± 4.4	<0.001
LAE * (%)	62 (28)	22 (52)	0.006
E/A ratio	0.92 ± 0.27	0.83 ± 0.21	0.004
E/e´	8.9 ± 2.6	11.6 ± 2.9	<0.001
DT (ms)	185 ± 33	212 ± 42	<0.001
LV DD (%)	116 (26)	41 (48)	<0.001

* -LAVI available for 263 patients (220 without events and 43 with events). LVEDD: left ventricle end-diastolic dimension; IVS: interventricular septum; RWT: relative wall thickness; LVM: left ventricle mass index; LVH: left ventricular hypertrophy; EF: ejection fraction; LAVI: left atrial volume index; LAE: left atrial enlargement; E: early diastolic mitral flow (pulse Doppler); A: late diastolic mitral flow (pulse Doppler); e´: early diastolic flow velocity across the lateral segment of mitral (e´) annulus (tissue Doppler); DT; deceleration time; LV DD: left ventricular diastolic dysfunction.

**Table 3:** Predictors of adverse events in the study population.

	Univariate Analysis			Multivariate Analysis		
	OR	95% CI	*p*	OR	95% CI	*p*
Model 1 (Dippers vs. non-dippers)						
Age (year)	1.11	1.03–1.85	0.014	0.84	0.64–1.51	0.147
Sex (male)	1.15	0.72–1.84	0.556	1.52	0.80–1.96	0.129
BMI (kg/m^2^)	1.09	0.82–1.94	0.091	0.84	0.70–1.92	0.143
Total cholesterol (mmol/L)	1.12	1.04–1.93	0.038	1.04	0.82–2.27	0.109
Serum creatinine (mmol/L)	1.07	0.92–1.52	0.104	1.03	0.71–2.06	0.208
Nighttime SBP (mmHg)	1.12	1.04–1.23	0.004	1.07	1.01–1.30	0.032
LVEF (%)	1.05	0.90–2.10	0.118	1.01	0.64–2.60	0.220
LVH (%)	2.65	1.57–4.48	<0.001	2.10	1.35–3.95	<0.001
LAE (%)	1.85	1.23–3.87	<0.001	-	-	-
LV DD (%)	2.61	1.62–4.18	<0.001	1.93	1.42–4.90	0.010
Dippers		Reference			Reference	
Non-dippers	2.27	1.41–3.66	<0.001	1.50	1.15–4.30	0.019
Model 2 (Dippers vs. non-dippers vs. reverse dippers vs. extreme dippers)						
Age (year)	1.11	1.03–1.85	0.014	0.80	0.57–2.46	0.172
Sex (male)	1.15	0.72–1.84	0.556	1.32	0.82–3.48	0.203
BMI (kg/m^2^)	1.09	0.82–1.94	0.091	0.92	0.48–2.89	0.258
Total cholesterol (mmol/L)	1.12	1.04–1.93	0.038	1.01	0.45–3.12	0.196
Serum creatinine (mmol/L)	1.07	0.92–1.52	0.104	1.05	0.55–2.68	0.310
Nighttime SBP (mmHg)	1.12	1.04–1.23	0.004	1.05	1.01–1.42	0.010
LVEF (%)	1.05	0.90–2.10	0.118	1.03	0.76–3.45	0.180
LVH (%)	2.65	1.57–4.48	<0.001	2.24	1.68–4.10	<0.001
LAE (%)	1.85	1.23–3.87	<0.001	-	-	-
LV DD (%)	2.61	1.62–4.18	<0.001	1.45	1.23–4.68	0.008
Dippers		Reference			Reference	
Non-dippers	1.92	1.09–3.36	0.023	1.30	0.98–4.25	0.075
Extreme dippers	0.76	0.31–1.83	0.672	0.70	0.56–1.57	0.551
Reverse dippers	2.61	1.34–5.09	<0.001	2.25	1.47–5.30	<0.001

ORL odd ratio; CI: confidence interval; BMI: body mass index; SBP: systolic blood pressure; LVEF: left ventricular ejection fraction; LVH: left ventricular hypertrophy; LAE: left atrial enlargement; LV DD: left ventricular diastolic dysfunction.

## Supplementary Materials

The following are available online at https://www.mdpi.com/2077-0383/8/12/2126/s1, Table S1: Demographic characteristics and clinical parameters of study population.
