# Supplementary material for: The Prognostic Effect of Circadian Blood Pressure Pattern on Long-Term Cardiovascular Outcome Is Independent of Left Ventricular Remodeling
Source: J Clin Med. 2019 Dec 2;8(12):2126. doi: 10.3390/jcm8122126 (PMC6947325; doi:10.3390/jcm8122126)
Supplement: Supplementary file 1 [file jcm-08-02126-s001.pdf]

Supplemental table 1. Demographic characteristics and clinical parameters of study population.

	Dippers (n=214)	Non-dippers (n=168)	Extreme dippers (n=77)	Reverse dippers (n=74)	p
**Age (years)**	52 ± 10	52 ± 11	50 ± 11	54 ± 12	0.158
**Male (%)**	112 (52)	92 (55)	40 (52)	41 (55)	0.406
**BMI (kg/m^2)**	26.7 ± 3.3	27.4 ± 3.9	26.5 ± 3.7	28.4 ± 4.2 [a,b]	0.002
**Adverse event (%)**	26 (12)	34 (20) [g]	7 (9) [d]	19 (26) [a,b]	0.006
**Clinic**					
**SBP (mmHg)**	152 ± 14	153 ± 15	150 ± 14	153 ± 14	0.456
**DBP (mmHg)**	93 ± 8	96 ± 9 [c]	93 ± 8 [d]	95 ± 9	0.003
**Heart rate (beat/min)**	77 ± 11	76 ± 9	75 ± 8	76 ± 10	0.453
**24-h**					
**SBP (mmHg)**	138 ± 14	139 ± 17	133 ± 16 [d]	137 ± 15	0.039
**DBP (mmHg)**	82 ± 9	83 ± 10	80 ± 9	81 ± 8	0.094
**Heart rate (beat/min)**	70 ± 9	71 ± 9	70 ± 8	72 ± 10	0.334
**Daytime**					
**SBP (mmHg)**	142 ± 14	140 ± 16	141 ± 15	137 ± 14	0.087
**DBP (mmHg)**	84 ± 11	85 ± 10	83 ± 9	80 ± 10 [e,f]	0.006
**Heart rate (beat/min)**	72 ± 9	73 ± 9	72 ± 8	74 ± 8	0.305
**Nighttime**					
**SBP (mmHg)**	124 ± 12	132 ± 14	109 ± 11	140 ± 16	<0.001*
**DBP (mmHg)**	77 ± 8	80 ± 9	71 ± 7	86 ± 9	<0.001*
**Heart rate (beat/min)**	61 ± 7	63 ± 8 [g]	61 ± 6	65 ± 7 [a,b]	<0.001

BMI – body mass index, SBP – systolic blood pressure, DBP – diastolic blood pressure.
* - p<0.01 for all comparisons; a- p<0.01 for dippers vs. reverse dippers; b- p<0.01 for extreme dippers vs. reverse dippers; c – p<0.01 for dippers vs. non-dippers; d- p<0.05 for non-dippers vs. extreme dippers; e- p<0.05 for dippers vs. reverse dippers; f – p<0.01 for non-dippers vs. extreme dippers; g- p<0.05 for dippers vs. non-dippers
